# MreC and MreD Proteins Are Not Required for Growth of *Staphylococcus aureus*

**DOI:** 10.1371/journal.pone.0140523

**Published:** 2015-10-15

**Authors:** Andreia C. Tavares, Pedro B. Fernandes, Rut Carballido-López, Mariana G. Pinho

**Affiliations:** 1 Laboratory of Bacterial Cell Biology, Instituto de Tecnologia Química e Biológica António Xavier, Universidade Nova de Lisboa, Oeiras, Portugal; 2 INRA, UMR 1319 Micalis, F-78350, Jouy-en-Josas, France; 3 AgroParisTech, UMR Micalis, F-78350, Jouy-en-Josas, France; ContraFect Corporation, UNITED STATES

## Abstract

The transmembrane proteins MreC and MreD are present in a wide variety of bacteria and are thought to be involved in cell shape determination. Together with the actin homologue MreB and other morphological elements, they play an essential role in the synthesis of the lateral cell wall in rod-shaped bacteria. In ovococcus, which lack MreB homologues, *mreCD* are also essential and have been implicated in peripheral cell wall synthesis. In this work we addressed the possible roles of MreC and MreD in the spherical pathogen *Staphylococcus aureus*. We show that MreC and MreD are not essential for cell viability and do not seem to affect cell morphology, cell volume or cell cycle control. MreC and MreD localize preferentially to the division septa, but do not appear to influence peptidoglycan composition, nor the susceptibility to different antibiotics and to oxidative and osmotic stress agents. Our results suggest that the function of MreCD in *S*. *aureus* is not critical for cell division and cell shape determination.

## Introduction

*Staphylococcus aureus* is a gram-positive pathogen responsible for many antibiotic-resistant hospital-acquired infections worldwide. It is well known for its capacity to cause various severe diseases such as bacteremia, pneumonia, endocarditis or osteomyelitis and for its increasing spread into the community [1]. Besides its clinical importance, *S*. *aureus* is also an excellent organism to study fundamental biological questions such as cell division and other cell cycle processes, given its simple shape and genetic tractability. Unlike the widely studied *Escherichia coli* and *Bacillus subtilis*, two rod-shaped model organisms, *S*. *aureus* is spherical and is therefore a good model to study morphogenesis of coccoid bacteria.

In most bacteria, a major factor contributing to the maintenance of cell shape is the presence of a cell wall outside the cytoplasmic membrane. The bacterial cell wall is generally composed of peptidoglycan, a mesh-like macromolecule made of glycan chains crosslinked by short peptide bridges. In gram-positive bacteria the peptidoglycan layer is thick (typically 30–100 nm), with proteins and anionic polymers embedded in it [2]. For bacterial cells to maintain a constant shape during growth and division, the activity of penicillin-binding proteins (PBPs), enzymes responsible for peptidoglycan synthesis, must be coordinated with the action of autolysins, which cleave peptidoglycan to allow cell wall expansion and splitting of the two daughter cells.

In rod-shaped bacteria, a second factor essential for the determination of cell shape is the presence of the MreB cytoskeleton. MreB-like proteins are structural homologues of eukaryotic actin that play an essential role in sidewall cylindrical elongation. These proteins have also been involved in other cellular processes like cell polarity and chromosome dynamics (reviewed in [3]). MreB homologues were proposed to associate in elongation-specific peptidoglycan-synthesizing complexes that effect lateral cell wall synthesis, together with other morphogenetic determinants, namely the transmembrane MreC and MreD proteins, as well as the presumed flippase RodA, PBPs and peptidoglycan hydrolases [4–7]. Recently, total internal reflection fluorescence microscopy (TIRFM) and high-precision particle tracking were used to show that, in *B*. *subtilis*, MreB isoforms co-localize with MreC, MreD, RodA and the co-essential transpeptidases PBPH and PBP2a, exhibiting circumferential processive motility [8,9]. Movement of these cell elongation machineries is driven by peptidoglycan synthesis itself, and is thought to be restricted and oriented by the underlying MreB filaments [3,8,9].

The roles of MreC and MreD on cell shape determination have been evidenced in different bacterial species. In elongated bacteria, like *B*. *subtilis*, *E*. *coli* and *Caulobacter crescentus*, absence of MreC and MreD results in growth arrest and severe morphological defects, as the cells become round, swelled or twisted, and eventually lyse [5,6,10–12]. Additionally, these membrane proteins were found to localize along the sidewall in a pattern similar to MreB [8–10,13] and to interact with peptidoglycan synthesizing enzymes. MreC was shown to interact not only with different PBPs [7,13,14] but also with the lytic transglycosylase MltA, the scaffolding protein MipA and some outer membrane proteins in *C*. *crescentus* [6,13]. Furthermore, MreD can interact with peptidoglycan biosynthesizing enzymes like MurG and MraY, and its presence is necessary for the correct localization of these proteins [6]. Although a specific function is not yet attributed to MreC and MreD, in rod-shaped bacteria these proteins are therefore thought to couple the internal bacterial cytoskeleton (MreB-like proteins) to the extracellular cell wall synthesizing complexes, coordinating sidewall elongation [7,10,11,13].

MreC is usually composed of one transmembrane domain near its N-terminal and a large C-terminal extracellular domain. In *C*. *crescentus*, however, this protein seems to be periplasmic [13]. MreD is a polytopic membrane protein predicted to have four to six transmembrane spans.

In coccoid cells, either spherical cocci (e.g staphylococci), or ovococci, with ellipsoid shape (e.g. streptococci), MreB proteins are in general absent but MreC and MreD are still present. Ovoccocci have not only septal peptidoglycan synthesis, but also peripheral peptidoglycan synthesis, responsible for the elongation of these cells, in which MreC and MreD are likely to play a role [15,16]. This is based on the fact that MreC and MreD localize to the equators and division septa of dividing *S*. *pneumoniae* cells and that MreCD-depletion in this organism leads to arrest of growth, cell rounding and lysis [15]. Interestingly, although MreC and MreD are essential for cell viability and cell shape maintenance in virulent strains D39 or TIGR4 [15,17], *mreCD* deletion mutants are viable and display normal growth and morphology in the *S*. *pneumoniae* laboratory strain R6 [15,18], which contains a suppressor mutation in *pbp1a* gene, encoding a PBP that influences the diameter of pneumococcal cells.

In spherical bacteria, such as *S*. *aureus*, cell wall synthesis occurs mainly at the division septa where the majority of PBPs are localized (reviewed in [19]), although some peptidoglycan synthesis activity is also detectable in the periphery of the cells, mediated mostly by PBP4 [20,21]. It is therefore intriguing why these organisms have retained MreC and MreD proteins, despite the lack of an MreB homologue and of a dedicated elongation machinery. While in *B*. *subtilis mreCD* genes are found immediately downstream of, and are co-transcribed with, *mreB* [22,23] and in *S*. *pneumoniae*, *mreCD* are found upstream of *pcsB*, a gene important for cell division in ovococcus, but apparently transcribed independently [18], in *S*. *aureus*, *mreCD* are not near any gene encoding identified division or morphology-related proteins.

In this work we show that MreC and MreD are not essential for viability of *S*. *aureus* cells as no growth, cell morphology or peptidoglycan synthesis defects were found in the absence of these proteins.

## Results and Discussion

### MreC and MreD are not required for *S*. *aureus* growth

**Fig 1:**
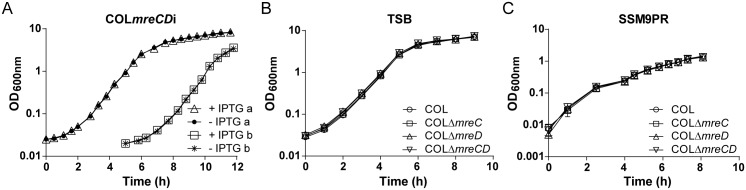
Depletion of MreC or MreD does not impair *S*. *aureus* COL growth. (A) Growth curves for inducible mutant COL*mreCD*i in TSB medium in the presence (Δ) and absence (●) of 0.5 mM IPTG (a). When the culture without IPTG reached OD_600nm_ ≈ 1, a sample was used to inoculate fresh media (b) with (□) and without (ӿ) 0.5 mM IPTG. (B and C) Growth curves for COL, COLΔ*mreC*, COLΔ*mreD* and COLΔ*mreCD* in TSB rich medium (B) and SSM9PR minimal medium (C). Mutant strains have a growth rate similar to the parental strain, with a duplication time of approximately 41 min in TSB and 81 min in SSM9PR.

MreC and MreD are essential for viability in the elongated bacteria studied so far and their absence results in growth arrest and lysis [5,10,12,15]. In order to test if *mreC* and *mreD* genes were also essential for the survival of spherical *S*. *aureus* cells, the *mreCD* operon was placed under control of the IPTG-inducible promoter P_*spac*_, by integration of the non-replicative plasmid pMutin4MreCt into the Methicillin Resistant *S*. *aureus* (MRSA) strain COL genome. To increase promoter repression in the absence of the inducer, the P_*spac*_ repressor LacI was expressed both from the integrated pMUTIN4 vector and the multicopy plasmid pMGPII [24]. We confirmed that expression of MreC in the resulting strain COL*mreCD*i was dependent on the presence of the inducer IPTG, although MreC protein levels were always below those expressed by the parental strain COL (S1 Fig). COL*mreCD*i had a similar growth rate, in rich TSB medium, in the presence and in the absence of the inducer (Fig 1A), suggesting that MreC and MreD are not essential proteins in *S*. *aureus* COL strain.

To confirm that *mreC* and *mreD* are not essential for *S*. *aureus* survival, we constructed *mreC*, *mreD* and *mreCD* deletion mutants in MRSA COL and in Methicillin Susceptible *S*. *aureus* (MSSA) strain NCTC8325-4, leaving the upstream and downstream regions of these genes intact and leaving no antibiotic marker. We were not able to delete the complete *mreD* gene from the genomes, even when an extra copy of *mreD* was expressed from the ectopic *spa* locus under the control of P_*spac*_ promotor. This may be due to polar effects on the expression of *rplU*, an essential gene [25] encoding the 50S ribosomal protein L21, which is located 166 bp downstream of *mreD* and oriented in the same direction. However, we could delete 374 bp (from nucleotide 7 to 380) of *mreD* (531 bp long) leaving the final 151 bp out of frame with the start codon, which resulted in strains COLΔ*mreD* and NCTCΔ*mreD*. Furthermore, we constructed strains COLΔ*mreCD* and NCTCΔ*mreCD* by deleting *mreC* and the first 380 nucleotides of *mreD*. The *mreC*, *mreD* and *mreCD* mutants were viable both in TSB rich medium and in SSM9PR minimal medium, and had similar growth rates relative to the COL and NCTC8325-4 parental strains (Fig 1 and S2 Fig). Importantly, although we did not see a growth defect for *mreCD* mutants, these genes are expressed in the laboratory conditions used, as shown by Western blotting of whole cell extracts of COL parental strain, grown in TSB rich medium, using an antibody against MreC (S3 Fig). We were not able to obtain an antibody against MreD, but the expression of an *mreD-sgfp* fusion from the native locus and controlled by the native promoter confirmed the expression of *mreD* gene (see below).

Suppressor mutations in laboratory strain R6 of *S*. *pneumoniae* allow the growth of *ΔmreCD* mutants [15]. In order to discard the hypothesis that lack of a growth phenotype in *S*. *aureus* Δ*mreC* and Δ*mreD* mutants was due to the acquisition of suppressor mutations, the entire genomes of COLΔ*mreC*, COLΔ*mreD* and COLΔ*mreCD* were sequenced, with a genome coverage of 99.99%, 99.93% and 99.86%, respectively. Deletion of *mreC* and *mreD* genes was confirmed in the respective strains and no mutations were found in COLΔ*mreC* when compared to parental strain COL. The genome of COLΔ*mreD* had only one SNP in SACOL1829, a gene of unknown function (S1 Table), which was intact in COLΔ*mreCD*. COLΔ*mreCD* had only one SNP in the plasmid recombination enzyme (pre) encoded in pT181, a natural staphylococcal plasmid present in strain COL. This protein is involved in plasmid maintenance and is unlikely to act as suppressor of *mreC* or *mreD* deletion. Importantly, none of the *S*. *aureus mreCD* mutants had any SNP in genes encoding PBPs, shown to suppress lack of *mreCD* in *S*. *pneumoniae* [15].

Thus, our results suggest that MreC and MreD are not essential proteins and do not play an important role in maintaining cell viability in *S*. *aureus* batch cultures.

### MreC and MreD localize mainly at the division septum

**Fig 2:**
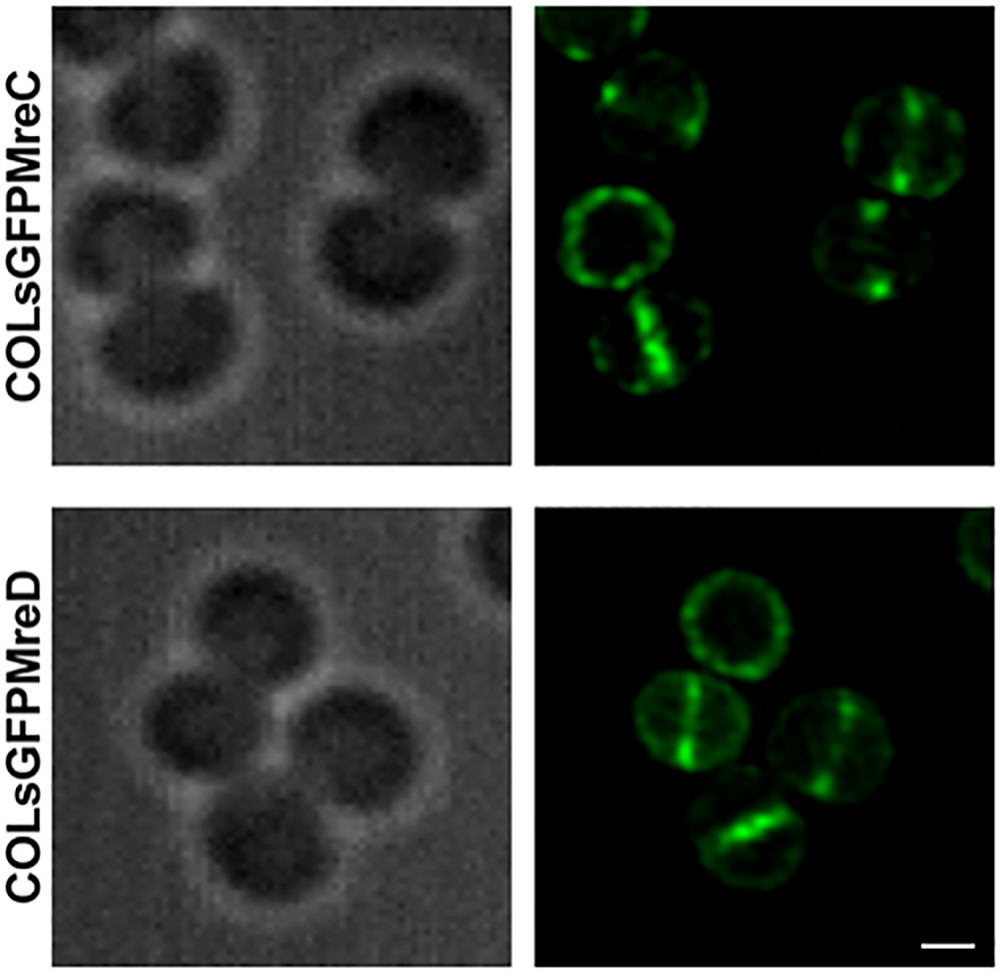
MreC and MreD are enriched at the division septum. *S. aureus* COL cells expressing sGFP fused to the N-terminal of MreC (top) or MreD (bottom) were grown in TSB supplemented with 0.2 mM IPTG and observed by SR-SIM. Left panels show differential interference contrast (DIC) images and right panels show fluorescence signal of sGFP fusion proteins. Scale bars, 0.5 μm.

In rod-shaped bacteria and in ovococci, MreC and MreD usually co-localize with the sidewall and peripheral cell wall synthetic machineries, respectively [6,7,13,14,16]. To study the localization of MreC and MreD in *S*. *aureus*, we constructed strains encoding superfast GFP [26] (sGFP) fused to the N-terminus of each protein. These fusions were expressed from an ectopic locus under control of the P_*spac*_ promoter. Superresolution Structured Illumination Microscopy (SR-SIM) and widefield fluorescence microscopy showed that both sGFP-MreC and sGFP-MreD localized mainly to the septa, with minor signal at the periphery of the cell (Fig 2 and S4A Fig). Septal enrichment of MreC and MreD proteins was analyzed using widefield fluorescence images by calculating the fluorescence ratio (FR) between the fluorescence at the centre of the division septum and the fluorescence at the peripheral cell membrane in cells with a complete septum [27] (S4A Fig). FR was 3.4 for sGFPMreC and 3.1 for sGFPMreD, confirming an enrichment of the two proteins at the division septum. In addition, *sgfp* was fused to the 3’ end of *mreD* at the native locus, replacing the native copy of *mreD*. This strain showed no morphology defects relative to the wild-type parental strain and natively expressed MreD-sGFP also displayed a preferential localization at the septum (S4B and S4C Fig).

### Deletion of *mreC* or *mreD* does not alter peptidoglycan synthesis

**Fig 3:**
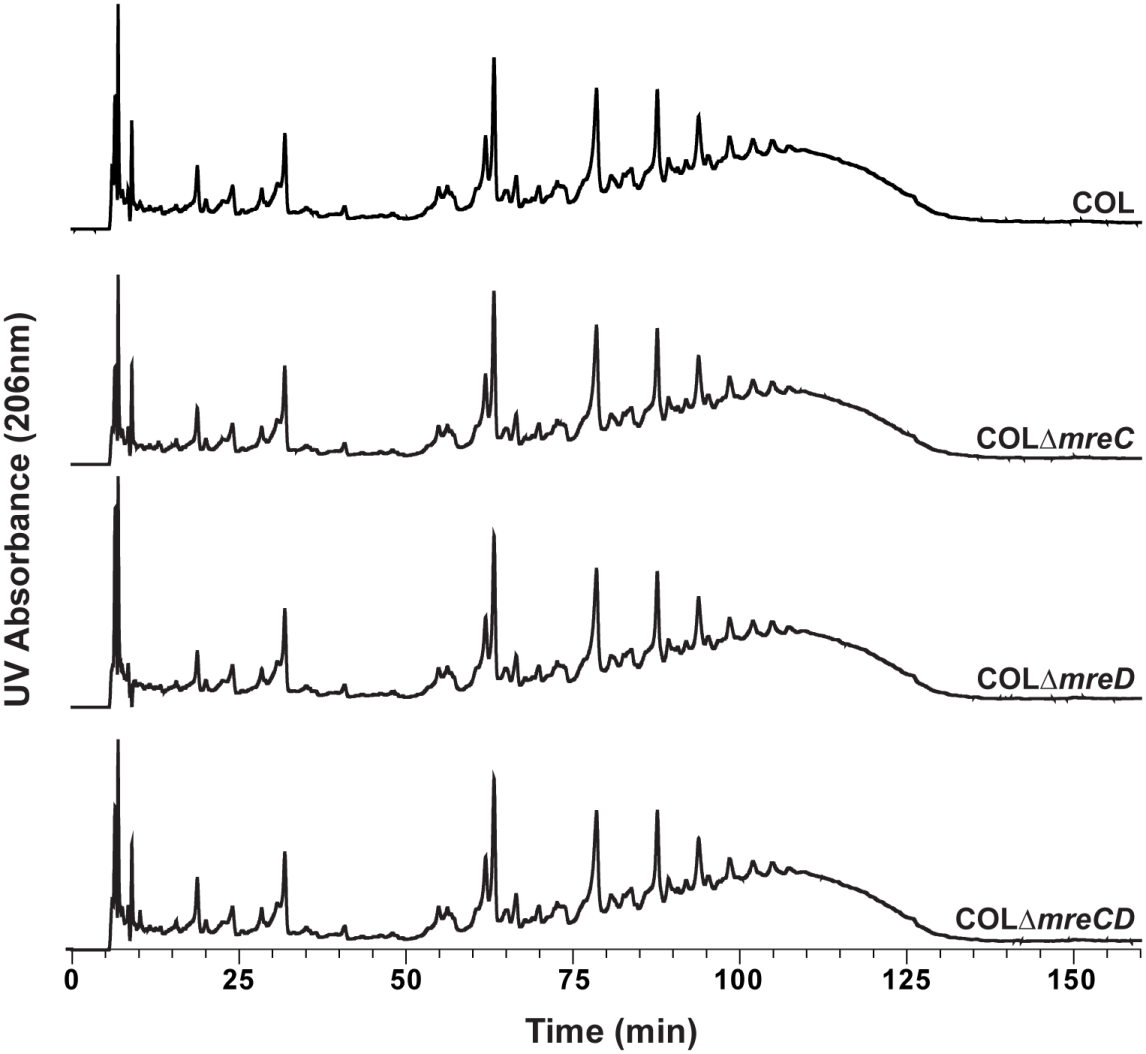
Profiles of peptidoglycan muropeptides remain unaltered in the absence of MreC or MreD. HPLC profiles of peptidoglycan muropeptides from *S*. *aureus* COL, COLΔ*mreC*, COLΔ*mreD* and COLΔ*mreCD*.

Localization of MreC and MreD to the septum, where peptidoglycan is mainly synthesized in *S*. *aureus* (reviewed in [19]), made us question about their possible role in maintaining cell wall composition. To test this, peptidoglycan from the parental strain COL and the Δ*mreCD* mutants was purified and digested with mutanolysin to cleave glycan strands. The resulting muropeptides were analyzed by reverse-phase high-performance liquid chromatography (HPLC). All the strains exhibited similar profiles (Fig 3), indicating that no significant alterations occur in peptidoglycan muropeptide composition or crosslinking in the absence of MreC and/or MreD.

It has been recently shown that a small fraction of peptidoglycan synthesis in *S*. *aureus* occurs at the cell periphery, mediated mainly by PBP4 [20,21]. Given that (i) MreC and MreD proteins are proposed to be involved in the synthesis of lateral cell wall in elongated bacteria, and (ii) we observed a small fraction of MreC and MreD localized at the cell periphery, we decided to further explore the possible role of these proteins in peripheral peptidoglycan synthesis.

For that purpose we investigated the sites of peptidoglycan incorporation in *mreC* and *mreD* mutants, as well as in the parental strain. Cells were labelled with NADA, a green fluorescent derivative of 3-amino-D-alanine that can be incorporated in the pentapeptide chain of peptidoglycan by PBPs [28,29]. In COL parental strain, incorporation of NADA occurred mostly at the division septum, but also, at lower levels, in the peripheral wall mainly in cells that are not synthesizing the septum (S5 Fig), as it had been previously shown [20,21]. The Δ*mreC* and Δ*mreD* mutants displayed the same pattern of peptidoglycan insertion (S5 Fig). The ratio between NADA signal at the septum and the periphery of the cell was similar for all the strains (S5A Fig), suggesting that peripheral cell wall synthesis is not dependent on MreC and/or MreD in *S*. *aureus*. Furthermore, COL and COLΔ*mreCD* were labeled with NADA and imaged in the same microscopy slide (previously labeling one or the other strain with DNA dye Hoechst 33342 to distinguish both strains) and fluorescence at the septum was determined. Lack of MreC and MreD did not impair septal incorporation of peptidoglycan, as values obtained for both strains were similar (S5B and S5C Fig). To confirm that the observed fluorescence signal was due to NADA incorporation and not to non-specific interaction of NADA with the cell surface, we labeled parental strain and COLΔ*mreCD* mutant cells with NADA L-enantiomer NALA, which is not incorporated into the peptidoglycan [28] and no signal was detected.

### Cell volume and morphology are maintained in *mreCD* deletion and overexpression mutants

**Fig 4:**
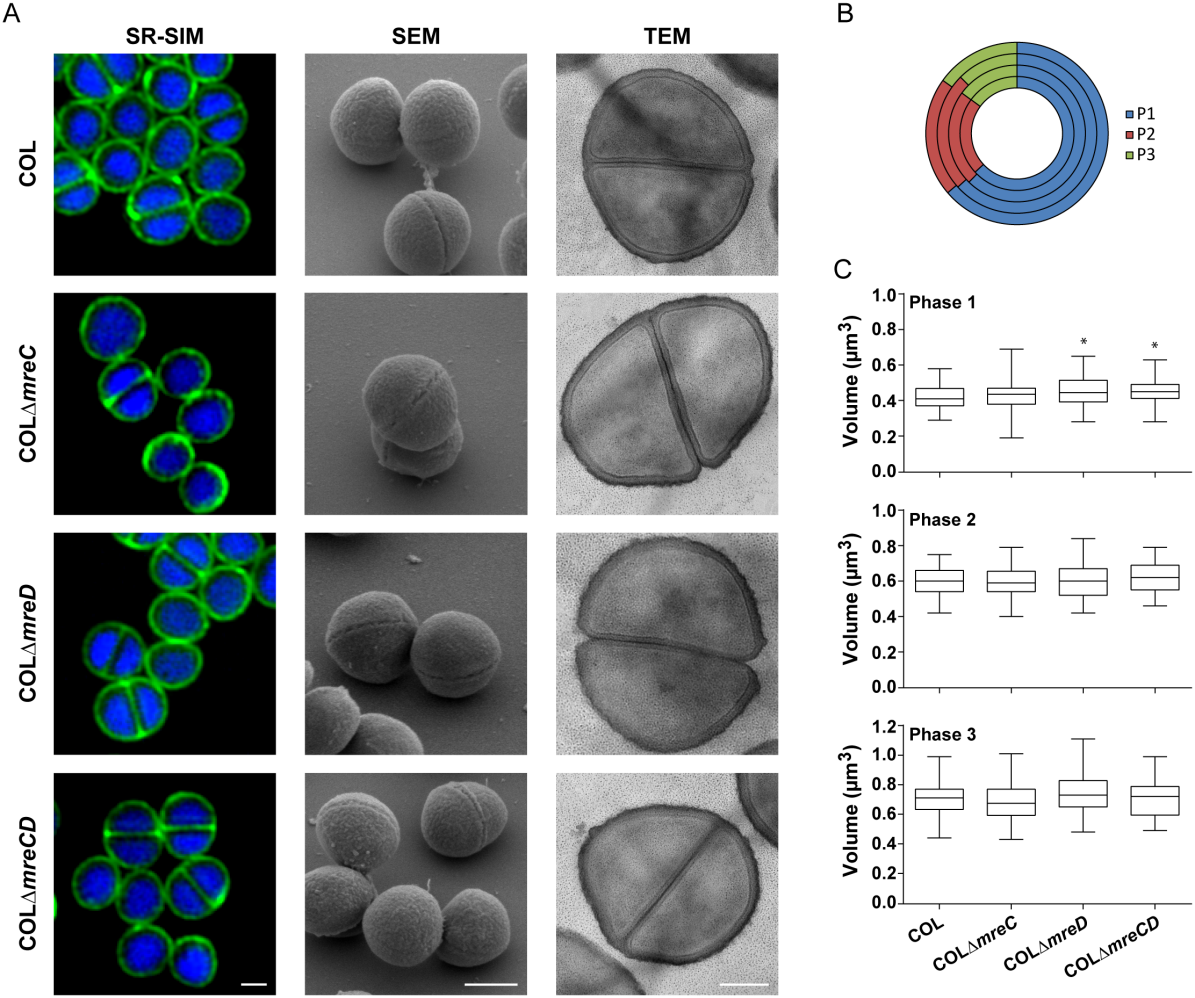
Absence of MreC or MreD has no effect on cell morphology, cell dimensions or cell cycle progression. (A) COL, COLΔ*mreC*, COLΔ*mreD* and COLΔ*mreCD* were imaged by Superresolution Structured Illumination Microscopy (SR-SIM), Scanning Electron Microscopy (SEM) and Transmission Electron Microscopy (TEM). For the SR-SIM images, cell wall and DNA were labeled with fluorescent vancomycin (Van-FL) and Hoechst 33342, respectively. Scale bars for SR-SIM and SEM images 0.5 μm and for TEM images 0.2 μm (B) Fraction of the cell cycle spent in Phase 1 (blue, before initiation of septum synthesis), Phase 2 (red, septum synthesis) and Phase 3 (green, after closure of the septum, before the two daughter cells split) [20]. Cell cycle progresses clockwise. From the inner to the outer circle the values correspond to COL, COLΔ*mreC*, COLΔ*mreD* and COLΔ*mreCD*. Exponentially growing cultures were labeled with the membrane dye Nile Red and the percentage of cells in each phase of the cell cycle was determined. (C) Cellular volume distributions during each phase of the cell cycle. Cells were labeled with Nile Red and imaged by SR-SIM. The volume was calculated by approximation of the cellular shape to a prolate spheroid. n = 60 for each phase. Statistical analysis was performed using the unpaired *t* test and p-values were >0.05 in all cases compared to the parental strain COL, except for Phase 1 COLΔ*mreD* (*p* = 0.013) and for COLΔ*mreCD* (*p* = 0.014).

In elongated bacteria, MreC and MreD are crucial to determine cell shape and when one of these two proteins is absent, cells become shorter and wider, acquiring a more spherical shape [5,6,10,12]. To test if MreC and MreD have some role in cell morphogenesis or division in *S*. *aureus*, the cell wall and DNA of deletion mutants and of COL parental strain were labeled using fluorescent vancomycin and Hoechst 33342, respectively, and imaged by SR-SIM. The same strains were also analyzed by Scanning and Transmission Electron Microscopy (SEM and TEM). As seen in Fig 4A, *mreC*, *mreD* and *mreCD* deletion mutants had no evident defects in DNA organization and maintained their spherical shape and surface roughness relative to the parental strain. Growth rate and cell morphology were also not affected by overexpression of MreC and MreD, as observed in strain COL harboring the replicative plasmid p*mreCD* which contains a copy of *mreCD* operon under the control of the inducible P_cad_ promoter (S6 Fig).

We next measured the volume of Δ*mreC* and Δ*mreD* cells at three different phases of the cell cycle. Phase 1 is the initial stage preceding division septum formation. Phase 2 corresponds to the formation of the division septum and Phase 3 is the final stage in which *S*. *aureus* cells have a complete septum before splitting into two daughter cells [20]. In *S*. *aureus*, impairment of cell division, for example due to depletion of FtsZ protein, results in cell enlargement [30]. Only minor differences were observed in the cell volume distributions between the Δ*mreCD* mutants and the parental strain (Fig 4C and S2 Table). Also, the relative time that each strain spent in each phase of the cell cycle remained approximately constant (Fig 4B), suggesting that MreC and MreD do not influence cell cycle progression and are not critical for cell division.

### Susceptibility to different stress agents is not affected by *mreC* or *mreD* deletion

As we were not able to establish a link between MreC and MreD and morphogenesis or peptidoglycan synthesis in standard laboratory growth conditions, we looked for alternative functions by assessing the susceptibility of the *mreCD* mutants to different stresses. We first tested resistance against antibiotics with different cellular targets including DNA replication (nalidixic acid), protein synthesis (chloramphenicol) and several steps of cell wall synthesis (phosphomycin, bacitracin, tunicamycin, D-cycloserine, oxacillin and vancomycin). The minimum inhibitory concentration (MIC) of Δ*mreC* and Δ*mreD* mutants to these compounds remained unaltered (S3 Table). Next, osmotic, oxidative and acidic stresses were induced by adding to the medium increasing concentrations of sodium chloride, hydrogen peroxide and hydrochloric acid, respectively. Again, no differences were observed between the susceptibility of the mutants and the parental strain (S3 Table). Taken together, these results indicate that MreC and MreD were not required to resist these stress conditions.

## Final Remarks

The morphogenetic elements MreC and MreD are essential proteins and play a key role in sidewall elongation in various rod-shaped and ovococcoid bacteria, in cylindrical and peripheral peptidoglycan synthesis respectively. Their presence in *S*. *aureus*, a spherical bacterium with no dedicated elongation machinery, raised the hypothesis they could be important for septal peptidoglycan synthesis, or be involved in the recently reported minor peptidoglycan synthesis activity at the cell periphery [20,21]. In this work we showed that *mreC* and *mreD* are not essential in MRSA strain COL and in MSSA strain NCTC8325-4 and do not affect cell morphology, cell volume or cell cycle progression in the laboratory growth conditions tested, in which we have shown that MreC and MreD are expressed. Peptidoglycan muropeptide composition and peripheral peptidoglycan incorporation also remained unaltered, indicating that MreCD do not play a major role in peptidoglycan synthesis in *S*. *aureus*. Although the lack of an important role in *S*. *aureus* for these proteins was unexpected, there are precedents in nature for cocci that do not use MreCD: *Streptococcus pyogenes* and *Streptococcus agalactiae*, two ovococcus species, do not encode recognisable homologues of MreC and MreD (reviewed in [31]).

It is possible that MreCD from elongated bacteria have two functions, one in cell shape/lateral peptidoglycan synthesis and a second, non-essential unknown function, which is the one conserved in spherical bacterial. Phenotypes resulting from the lack of this second function may be visible only in conditions that were not covered in this study, which differ considerably from growth in natural environments. Further work is necessary to understand the possible roles of these two proteins in *S*. *aureus*.

## Materials and Methods

### Bacterial strains, plasmids and growth conditions

The plasmids and bacterial strains used in this study are described in Tables A and B in S1 File. *S*. *aureus* strains were grown at 37°C in Tryptic soy broth (TSB; Difco), Tryptic soy agar (TSA; Difco) or in SSM9PR minimal media [32] containing 1 x M9 salts (12.8 g/L Na_2_HPO_4_, 3 g/L KH_2_PO_4_, 5 g/L NaCl, 1 g/L NH_4_Cl), 2 mM MgSO4, 0.1 mM CaCl2, 1% glucose, 1% casaminoacids, 1 mM Thiamine-HCl and 0.05 mM nicotinamide. Culture medium was supplemented with appropriate antibiotics (10 μg/ml erythromycin, 10 μg/ml chloramphenicol or 150 μg/ml kanamycin; Sigma-Aldrich), with 100 μg/ml 5-bromo-4-chloro-3-indolyl-β-D-galactopyranoside (X-Gal; VWR), with isopropyl-β-D-thiogalactopyranoside (IPTG; VWR) or with 0.5 mM CdCl_2_, when required. *E*. *coli* strains were grown at 37°C in Luria-Bertani broth (LB; Difco) or in LB agar (Difco) supplemented with 100 μg/ml ampicillin (Sigma-Aldrich) when necessary.

### Construction of S. *aureus* strains

Primers used in this study are listed in Table C in S1 File. The *mreCD* inducible mutant containing the *mreCD* operon under the control of IPTG-inducible P_spac_ promoter was constructed using the integrative vector pMUTIN4 [33]. A fragment containing the putative RBS and the first 463 bp of *mreC* gene was amplified by PCR from *S*. *aureus* COL genomic DNA using primers MreCt-P1 and MreCt-P2. The fragment was digested with *Hind*III and *Bam*HI restriction enzymes, cloned into pMUTIN4 vector in *E*. *coli*, sequenced, electroporated into *S*. *aureus* RN4220 strain at 30°C, using erythromycin selection, and transduced into *S*. *aureus* COL using phage 80α [34]. The *mreCD* operon was placed under the control of the P_spac_ promoter through a single crossover event. To increase the level of repression of the P_spac_ promoter, the pMGPII multi-copy plasmid [24] encoding an extra *lacI* gene was also transduced into this strain. All procedures were performed in the presence of 0.5 mM IPTG. The integration of the plasmid into the *mreC* chromosomal locus of the final strain COL*mreCD*i strain was confirmed by PCR amplification.

Deletion of *mreC*, *mreD* and *mreCD* from the genome was performed using the thermosensitive plasmid pMAD [35]. The deletion mutants were constructed by PCR amplification, from *S*. *aureus* COL genomic DNA, of the upstream and downstream regions of *mreC* (dMreC-P1/dMreC-P2 and dMreC-P3/dMreC-P4), *mreD* (dMreD-P1/dMreD-P2 and dMreD-P3/dMreD-P4) and *mreCD* (dMreC-P1/dMreCD-P2 and dMreCD-P3/dMreD-P4). The upstream and downstream fragments of *mreC*, *mreD* and *mreCD* were joined by overlap PCR using primers dMreC-P1/dMreC-P4, dMreD-P1/dMreD-P4 and dMreC-P1/dMreD-P4, respectively. The resulting fragments were digested with *Xma*I and *Bam*HI restriction enzymes and cloned into pMAD vector producing the plasmids pΔ*mreC*, pΔ*mreD* and pΔ*mreCD*. The inserts were sequenced and the plasmids were then electroporated into *S*. *aureus* RN4220 strain at 30°C, using erythromycin and X-gal selection, and transduced into *S*. *aureus* COL and NCTC8325-4 using phage 80α. Integration and excision of the plasmids into the chromosome was performed as previously described [35], resulting in strains COLΔ*mreC*, COLΔ*mreD*, COLΔ*mreCD*, NCTCΔ*mreC*, NCTCΔ*mreD* and NCTCΔ*mreCD*. Gene deletions were confirmed by PCR and sequencing.

For the overexpression of MreC and MreD, a fragment containing the entire *mreCD* operon was amplified using primers MreC_*Sma*I_P1 and MreD_*Eco*RI_P2. The fragment was digested with *Sma*I and *Eco*RI restriction enzymes and cloned into pCNX vector [20] in *E*. *coli*. After sequencing, the plasmid was electroporated into *S*. *aureus* RN4220 strain at 37°C, using kanamycin selection, and transduced into *S*. *aureus* COL using phage 80α resulting in strain COLp*mreCD*. As a control, empty vector pCNX was transduced into *S*. *aureus* COL strain resulting in strain COLpCNX.

To localize MreC and MreD proteins, N-terminal fusions with the P7 variant of superfast GFP [26] (sGFP) were constructed. A fragment containing a functional RBS sequence, the 714 bp *sgfp* gene excluding the stop codon and a sequence encoding a 5 amino acid linker was amplified by PCR from pTRC99a-P7 plasmid [26] using the primers sGFP-*Sma*I-P1 / sGFPMreC-P2 (*mreC* fusion) and sGFP-*Sma*I-P1/ sGFPMreD-P2 (*mreD* fusion). Primers sGFPMreC-P3 / sGFPMreC-*Xho*I-P4 and sGFPMreD_P3 / sGFPMreD-*Xho*I-P4 were used to amplify *mreC* and *mreD* genes, respectively. The fragments were joined by overlap PCR using the primers sGFP-*Sma*I-P1 / sGFPMreC-*Xho*I-P4 and sGFP-*Sma*I-P1 / sGFPMreD-*Xho*I-P4. The resulting fragments were digested with *Sma*I and *Xho*I restriction enzymes and cloned into the pBCB13 vector [36]. The plasmids were electroporated into *S*. *aureus* RN4220 at 30°C using erythromycin and X-gal selection and then transduced into *S*. *aureus* strains COL using phage 80α. By consecutive integration and excision, the *sgfp* fusions were introduced in the *spa* locus by replacement of the *spa* gene, giving rise to the final strains COLsGFPMreC and COLsGFPMreD. The localization of MreD was also achieved by replacement of the native *mreD* gene by a *mreD-sgfp* fusion in the native locus. For this purpose, integrative plasmid pFAST2 was constructed by amplifying a 809 bp fragment encompassing the coding region of *sgfp* from pTRC99a-P7 using the primers sGFP-EcoRV-P1 and sGFP-NotI-P2, digested with *Eco*RV and *Not*I (Fermentas), and used to replace the *gfpmut*P2 gene in pSG5082 [37]. Subsequently, the 483 final nucleotides of *mreD* excluding the stop codon, were amplified by PCR using the primers MreDsGFP-*Hind*III-P1 and MreDsGFP-*Bam*HI-P2 and cloned into pFAST2, in frame with the *sgfp* gene. The final plasmid pFAST2MreDsGFP was electroporated into *S*. *aureus* RN4220 and then transduced into *S*. *aureus* COL. The integration of this plasmid in the native *mreD* locus occurred through a single crossover event and was confirmed by PCR. The final strains were named COLpFAST2MreDsGFP.

### Genome Sequencing

Genomic DNA was extracted from COL parental strain and *mreCD* deletion mutants and sequenced using the Illumina MiSeq system (Instituto Gulbenkian de Ciência, Oeiras, Portugal). 250-bp paired end reads with 100x average coverage were generated. Sequence reads were then assembled and analyzed with SeqMan NGen^®^ and SeqMan Pro^®^ (Version 12.0. DNASTAR. Madison, WI) software. COL genome (NCBI Accession NC_002951.2) was used as a reference template. The specific variations of the deletion mutants were identified by comparison with the respective parental strain. Low quality variations with read frequencies below 50% were removed from the dataset.

### Growth curves of *S*. *aureus* strains.

Growth of *mreCD* inducible mutants was analyzed by growing the cells overnight at 37°C in TSB medium supplemented with appropriate antibiotics and 0.5 mM IPTG. The overnight culture was washed four times with TSB and diluted 1/500 into fresh TSB medium with (control) or without 0.5 mM IPTG. When the culture without IPTG reached an OD_600nm_ of approximately 1, a 1 ml sample was taken, washed four times and diluted 1/500 into fresh TSB medium with and without 0.5 mM IPTG. All cultures were incubated at 37°C with agitation and the OD_600nm_ was recorded. For the growth of the deletion mutants the TSB overnight cultures were diluted to an initial OD_600nm_ of 0.002 in fresh medium (TSB or SSM9PR minimal medium), in triplicate. Cultures were incubated at 37°C with shaking and OD_600nm_ was monitored.

### Protein Purification and antibody production

The truncated *mreC* gene lacking the DNA fragment encoding the N-terminal transmembrane region was amplified by PCR from COL genomic DNA using primers MreCprot-P1 and MreCprot-P2. The resulting fragment was digested with *Bam*HI and *Xho*I restriction enzymes and cloned into the pET30a vector. The resulting plasmid, pETMreCt, encodes a combined His_6_-tag and S-tag N-terminal fusion of the truncated MreC. pETMreCt was introduced into *E*. *coli* BL21 (DE3) by heat-shock transformation. The transformants were grown at 37°C in LB medium containing 50 μg/ml Kanamycin. At OD_600nm_ of approximately 1, the cultures were supplemented with 1 mM IPTG and grown for 3 additional hours. Cells were harvested by centrifugation and re-suspended in buffer A (50 mM sodium phosphate buffer pH 8 containing 150 mM NaCl, and complete-EDTA-free protease inhibitors [Roche]). The cells were incubated at 4°C for 15 min in the presence of DNase (10μg/ml), RNase (20μg/ml) and lysozyme (0.4 μg/ml) and then disrupted by sonication. The samples were centrifuged at 16000 rpm at 4°C. As most of the protein was in the pellet (confirmed by 12% SDS-PAGE), the pellet was resuspended in buffer B (50 mM sodium phosphate buffer pH 8 containing 300 mM NaCl and 0.1% Brij) supplemented with 8 M Urea. The suspension was applied to pre-equilibrated Talon^TM^ (Clontech) resin and incubated at 4°C for 48h. The same volume of buffer B without urea was added to the sample, to reduce the concentration of urea to 4 M, and the suspension was incubated at 4°C overnight. Bound protein was washed once with buffer B with 4 M urea followed by two washes without urea. Two sequential elutions with elution buffer (50 mM sodium phosphate buffer pH 8, 300 mM NaCl) containing 100 mM and 150 mM imidazole respectively were performed. Eluted fractions were mixed and dialyzed in three consecutive steps against 50 mM sodium phosphate buffer (pH 8) containing 500 mM, 300 mM and 150 mM NaCl, respectively. The purified protein was digested with enterokinase (New England Biolabs) to cleave His_6_-tag and S-tag and separated on a 12% SDS-PAGE. The band corresponding to the truncated MreC was cut from the gel and sent to Eurogentec (Belgium) for polyclonal antibody production.

### Western Blotting

*S*. *aureus* strains were grown overnight, diluted 1/500 in fresh medium and incubated at 37°C. When necessary, cultures were supplemented with 0.5 mM IPTG or 0.5 mM CdCl_2_. At OD_600nm_ of approximately 0.6 cells were harvested and broken with glass beads in a FastPrep FP120 cell disrupter (Thermo Electro Corporation). Samples were centrifuged to remove unbroken cells and debris and total protein content of the clarified lysates was determined using the Bradford method and bovine serum albumin as a standard (BCA Protein Assay Kit, Pierce). Equal amounts of total protein from each sample were separated on 12% SDS-PAGE at 120V and then transferred to Hybond-P PVDF membrane (GE Healthcare) using a BioRad Semi-dry transfer cell, according to standard western blotting techniques. MreC and PBP2 proteins were detected using specific polyclonal antibodies.

### Scanning electron microscopy

Exponentially growing *S*. *aureus* cells were harvested by centrifugation, resuspended in fixative solution (2.5% glutaraldehyde in 0.2 M sodium cacodylate buffer, pH 7.4), deposited on glass discs (Marienfeld) and kept for 1 week at 4°C. The fixative solution was subsequently removed and the cells were washed three times with the sodium cacodylate solution. The sample was progressively dehydrated by immersion in a graded series of ethanol (50% - 100%) and then mounted on aluminum stubs with carbon adhesive discs (Agarscientific). The sample was critical-point dried under CO_2_ and sputter coated with gold-palladium (Polaron SC7640) for 200s at 10mA. SEM observations were performed using secondary electron images (2 kV) with a Hitachi S4500 instrument at the Microscopy and Imaging Platform (Micalis, Massy, France) of the INRA research center of Jouy-en-Josas (France).

### Transmission electron microscopy

Exponentially growing *S*. *aureus* cells were harvested by centrifugation and fixed with 2% glutaraldehyde in 0.1 M Na cacodylate buffer pH 7.2, for 3 hours at room temperature. Cells were then contrasted with Oolong Tea Extract (OTE) 0.5% in cacodylate buffer and postfixed with 1% osmium tetroxide containing 1.5% potassium cyanoferrate. Samples were gradually dehydrated in ethanol (30% to 100%), substituted progressively in a mix of propylene oxyde-epon and embedded in Epon (Delta microscopie—Labège France). Thin sections (70 nm) were collected onto 200 mesh cooper grids, and counterstained with lead citrate. Grids were examined with Hitachi HT7700 electron microscope operated at 80kV and images were acquired with a charge-coupled device camera (AMT). Electron microscopy has benefited from the facilities and expertise of MIMA2 MET, INRA (France).

### Determination of the susceptibility to stress agents

The minimum inhibitory concentrations (MICs) of *S*. *aureus* strains to antibiotics, to oxidative stress agent hydrogen peroxide (H_2_O_2_) and to osmotic stress agent sodium chloride (NaCl) were determined by microdilution in 96-well plates. The wells contained serial two fold dilutions of each compound in a total volume of 100 μl of TSB. For the antibiotic susceptibility assay each well was individually inoculated with 5 μl of a 10^−3^ dilution of an overnight culture. For the oxidative and osmotic susceptibility assays TSB medium was inoculated to a final OD_600nm_ of 0.02. A sterility control was performed with only TSB. Plates were incubated at 37°C without shaking for 24h or 48h and the MICs were recorded as the lowest concentration of compound that inhibited bacterial growth.

Higher acidic pH value which inhibits cell growth was determined in a similar way using hydrochloric acid (HCl).

### Peptidoglycan purification and analysis

*S*. *aureus* parental and mutant strains were grown in TSB until OD_600nm_ of 0.9 and peptidoglycan was purified from exponentially growing cells as previously described [38]. Muropeptides were prepared by digestion with mutanolysin and analyzed by reverse phase HPLC using a Hypersil ODS (C18) column (Thermo Fisher Scientific). Muropeptide species were eluted in 0.1 M sodium phosphate, pH 2.0, with a gradient of 5–30% methanol for 155 min and detected at 206 nm.

### Labeling and imaging of *S*. *aureus*

Strains with *sgfp* fusions were grown overnight, diluted 1/200 in fresh medium and incubated at 37°C. 0.2 mM IPTG was added to the cultures expressing the fusions from the ectopic *spa* locus. At OD_600nm_ of approximately 0.6, 1 ml of each culture was centrifuged, resuspended in phosphate-buffered saline (PBS) and 1 μl drop was placed onto a thin film of 1.2% agarose prepared in PBS mounted on a microscopy slide.

Parental and deletion mutant strains were grown overnight, diluted 1/200 in fresh medium and incubated at 37°C. At OD_600nm_ of 0.5–0.7, 1 ml of each culture was taken and incubated for 5 min with DNA dye Hoechst 33342 (3 μg/ml, Invitrogen), membrane dye Nile Red (10 μg/ml, Invitrogen) or cell wall dye Vancomycin (Sigma) mixed with a BODIPY FL conjugate of vancomycin (Van-FL, Molecular Probes) to a final concentration of 0.8 μg/mL. The cells were then pelleted, resuspended in PBS and 1 μl of this cell suspension was placed onto a thin film of 1.2% agarose prepared in PBS mounted on a microscopy slide.

To localize the sites of peptidoglycan synthesis, cells were grown in TSB to an OD_600nm_ of approximately 0.6, labeled with fluorescent D-amino acid NADA or L-amino acid NALA (500 μM) [28,29] for 5 min at 37°C, 600 rpm and washed with TSB. The cells were resuspended in PBS and 1 μl drop of this cell suspension was placed onto a thin film of 1.2% agarose prepared in PBS and visualized by SR-SIM. To compare absolute NADA fluorescence in two different strains in the same microscopy image, one of them was labeled with DNA dye Hoechst 33342. The cells were then labeled with NADA, washed and mixed prior to visualization by widefield fluorescence microscopy and SR-SIM.

### Superresolution Structured Illumination Microscopy (SR-SIM)

SR-SIM imaging was performed using a Plan-Apochromat 63x/1.4 oil DIC M27 objective, in an Elyra PS.1 microscope (Zeiss). Images were obtained using either 3 or 5 grid rotations, with 34μm grating period for the 561nm laser, 28μm period for 488nm laser and 23μm period for 405nm laser. Images were acquired using a sCMOS camera and reconstructed using ZEN software (black edition, 2012, version 8.1.0.484) based on a structured illumination algorithm [39].

### Determination of cell cycle phase and cellular volume

The cell cycle phase and the cellular volume were determined based on SR-SIM images of *S*. *aureus* cells labeled with the membrane dye Nile Red. As previously described, the relative number of cells in one phase of the cell cycle is proportional to the percentage of time that those cells spend in that same phase [20]. To calculate the volume of each cell, an ellipse was overlaid with the membrane dye signal and fitted to the border limits of the cellular membrane. The shorter and longer axes of the ellipse were measured and the volume of the cell was calculated by an approximation to the volume of a prolate spheroid $\left(V=\frac{4}{3}\pi \;ab^{2}\right)$, where *a* and *b* correspond to the longer and shorter semi-axes, respectively. The volume was determined for a total of 60 cells in each phase of each strain.

### Fluorescence ratio determination

Cells expressing *sgfp*-*mreC*/*D* fusions and cells labeled with NADA were observed with a Zeiss Axio Observer microscope and images were taken with a Photometrics CoolSNAP HQ2 camera (Roper Scientific) using Metamorph 7.5 software (Molecular Devices). To determine the fluorescence ratio (FR), the fluorescence at the centre of division septum (only from cells with closed septa) was quantified and divided by the fluorescence at the peripheral cell wall. Average background fluorescence was subtracted from both values [27]. A total of 70 cells from each strain was analyzed.

### Statistical analysis

GraphPad Prism 6 (GraphPad Software) was used to perform the statistical analysis. The differences in cellular volume between each of the mutants and the parental strain and the comparisons between NADA-labeling fluorescence ratios were evaluated by unpaired student’s t-tests. p-values ≤ 0.05 were considered significant for all analysis performed and were indicated with asterisks: *p ≤ 0.05, **p ≤ 0.01 and ***p ≤ 0.001.

## Supporting Information

S1 Fig Detection of MreC in COL*mreCD*i inducible mutant. Western blot analysis of total protein extracts of COL, COLΔ*mreCD* and COL*mreCD*i grown in the absence or presence of IPTG inducer, using an MreC-specific antibody. In the inducible COL*mreCD*i mutant, MreC protein is not detected in the absence of the inducer and is detected in its presence, albeit in lower concentration than in the parental strain, even when an excess of IPTG is used. Detection of PBP2 (upper panel) was used as internal loading control.
(TIF)

S2 Fig Depletion of MreC or MreD does not affect *S*. *aureus* NCTC8325-4 growth. (A and B) Growth curves for NCTC, NCTCΔ*mreC*, NCTCΔ*mreD* and NCTCΔ*mreCD* in TSB (A) and SSM9PR (B) medium.
(TIF)

S3 Fig MreC is present in COL but not in COLΔ*mreC* nor COLΔ*mreCD*. Western blot analysis of total protein extracts of COL, COLΔ*mreC* and COLΔ*mreCD* using an anti MreC-specific antibody. MreC was present in the parental strain but was undetectable in the mutants.
(TIF)

S4 Fig MreC and MreD are enriched at the division septum. (A) Widefield fluorescence microscopy images of sGFPMreC and sGFPMreD fusions in *S*. *aureus* COL. Fluorescence ratio (FR) between the values quantified at the center of the division septum and the fluorescence at the peripheral cell membrane was calculated. A total of 70 cells with closed septa was analyzed for each strain. An FR above two indicates protein enrichment at the septum. (B-C) SR-SIM (B) and Widefield fluorescence microscopy (C) images of COLpFAST2MreDsGFP which expresses MreD-sGFP fusion from the native locus. Scale bars, 1 μm.
(TIF)

S5 Fig Peptidoglycan incorporation pattern in *S*. *aureus* is not altered in the absence of MreC and MreD. (A) COL, COLΔ*mreC*, COLΔ*mreD* and COLΔ*mreCD* were imaged by widefield fluorescence microscopy after being labeled for 5 minutes with fluorescent derivative of 3-amino-D-alanine, NADA. Incorporation of NADA occurs mainly at the septum but also in the peripheral cell wall. Fluorescence ratio (FR) between the values quantified at the center of the division septum and the fluorescence at the lateral cell wall was calculated. A total of 50 cells with closed septa were analyzed for each strain. The ratios were similar between COL (8.00±3.01) and the mutants Δ*mreC* (7.47±2.54, *p* = 0.3445), Δ*mreD* (8.69±2.62, *p* = 0.2262) and Δ*mreCD* (8.63±2.71, *p* = 0.2754). Scale bar, 1 μm. (B) Septal fluorescence (Septum) and fluorescence ratio (FR) for COL and COLΔ*mreCD*. Cells were labeled with NADA for 5 minutes, mixed together and observed by widefield fluorescence microscopy. To distinguish the two strains, one of them was previously labeled with the DNA dye Hoechst 33342 (shown in blue). The experiment was repeated exchanging the strain with labeled DNA to confirm that Hoechst 33342 labeling does not affect NADA fluorescence. No statistically significant differences were observed between COL parental strain and COLΔ*mreCD* mutant (*p*>0.05 for all conditions) (C) SR-SIM of COL parental strain (labeled with NADA and Hoechst 33342) and COLΔ*mreCD* (labeled only with NADA). Scale bar, 1 μm.
(TIF)

S6 Fig Overexpression of MreC and MreD does not affect *S*. *aureus* COL growth or morphology. A) Western blot analysis of total protein extracts of COL grown without (1) and with (2) 0.5 mM CdCl_2_, COLΔ*mreCD* (3), COLpCNX (empty vector) grown without (4) and with (5) 0.5 mM CdCl_2_ and COLp*mreCD* grown without (6) and with (7) 0.5 mM CdCl_2_. MreC detection was performed using an MreC-specific antibody. Overexpression of MreC is observed in COLp*mreCD* in the presence of the inducer (lane 7). PBP2 was used as internal loading control. (B, C) Growth curves (B) and SR-SIM (C) of COL, COLpCNX grown with 0.5 mM CdCl_2_ and COLp*mreCD* grown without and with 0.5 mM CdCl_2_. Scale bar, 1 μm. Growth curves and morphology of COLpCNX (parental strain with empty vector) and COLp*mreCD* (overexpressing MreCD), both grown in the presence of cadmium, are indistinguishable.
(TIF)

S1 File Includes supporting Tables A-C. Information about plasmids, strains and primers used in this study.
(DOCX)

S1 Table Genomic mutations in COLΔ*mreD* and COLΔ*mreCD* identified by whole genome sequencing by comparison with parental strain COL. (DOCX)

S2 Table Average volume (μm^3^) of *S*. *aureus* COL, COLΔ*mreC*, COLΔ*mreD* and COLΔ*mreCD* cells at the three phases of the cell cycle. (DOCX)

S3 Table Susceptibility to antibiotics, osmotic, oxidative and acidic stress agents of COL, COLΔ*mreC*, COLΔ*mreD* and COLΔ*mreCD*. Minimum Inhibitory Concentrations (MICs) of different antibiotics, sodium chloride and hydrogen peroxide and the higher acidic pH value which inhibits cell growth. The values in the table correspond to the results obtained for all the deletion mutants and the parental strain, after 24h incubation, except for oxacillin (48h).
(DOCX)
