# Supplementary figures and images for: MreC and MreD Proteins Are Not Required for Growth of Staphylococcus aureus
Source: PLoS One. 2015 Oct 15;10(10):e0140523. doi: 10.1371/journal.pone.0140523 (PMC4607420; doi:10.1371/journal.pone.0140523)

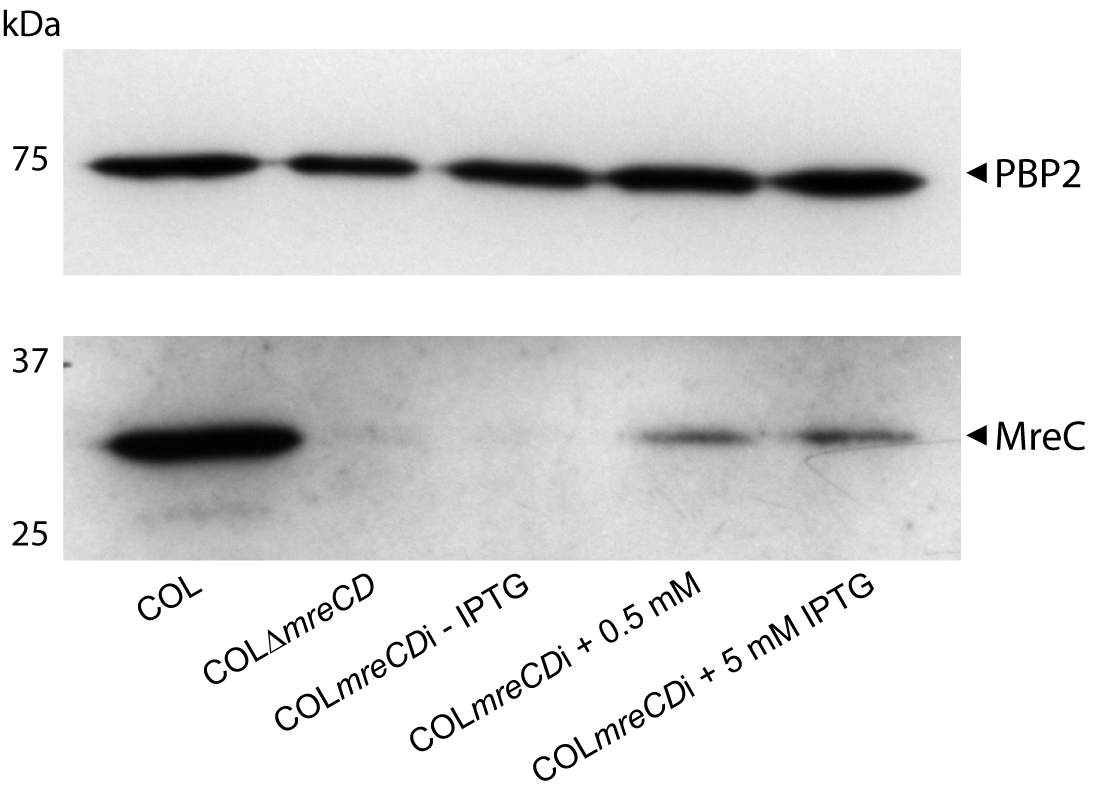

Supplement: S1 Fig — Western blot analysis of total protein extracts of COL, COLΔmreCD and COLmreCDi grown in the absence or presence of IPTG inducer, using an MreC-specific antibody. In the inducible COLmreCDi mutant, MreC protein is not detected in the absence of the inducer and is detected in its presence, albeit in lower concentration than in the parental strain, even when an excess of IPTG is used. Detection of PBP2 (upper panel) was used as internal loading control. (TIF) [file pone.0140523.s001.tif]

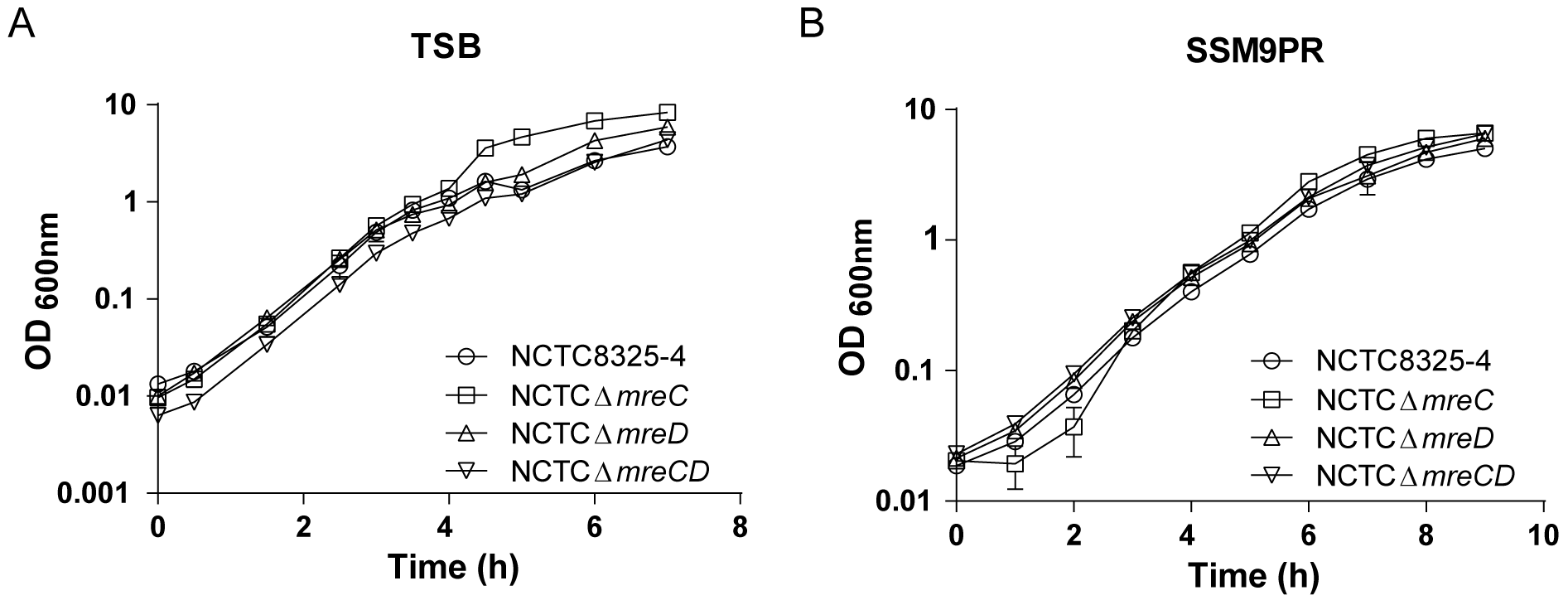

Supplement: S2 Fig — (A and B) Growth curves for NCTC, NCTCΔmreC, NCTCΔmreD and NCTCΔmreCD in TSB (A) and SSM9PR (B) medium. (TIF) [file pone.0140523.s002.tif]

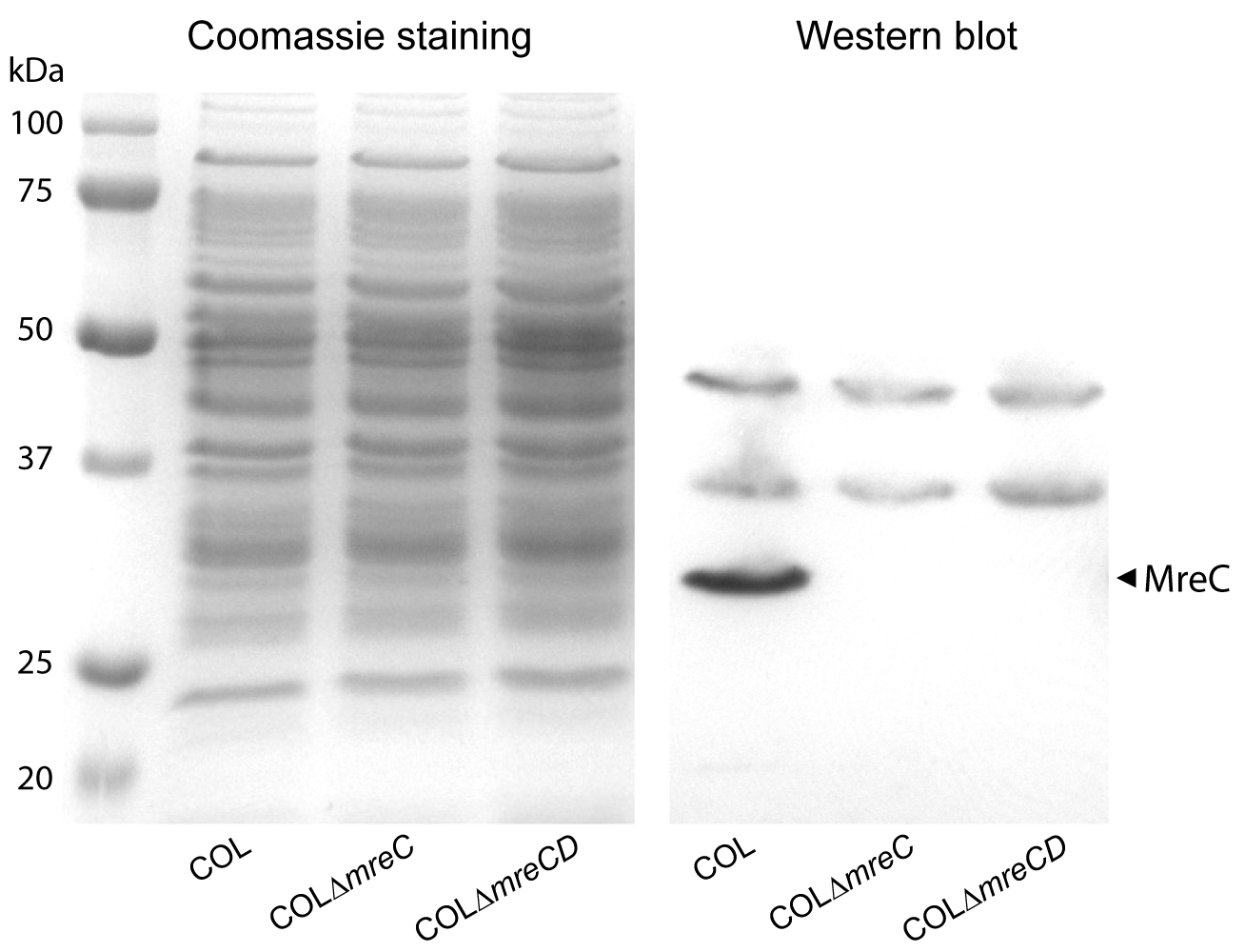

Supplement: S3 Fig — Western blot analysis of total protein extracts of COL, COLΔmreC and COLΔmreCD using an anti MreC-specific antibody. MreC was present in the parental strain but was undetectable in the mutants. (TIF) [file pone.0140523.s003.tif]

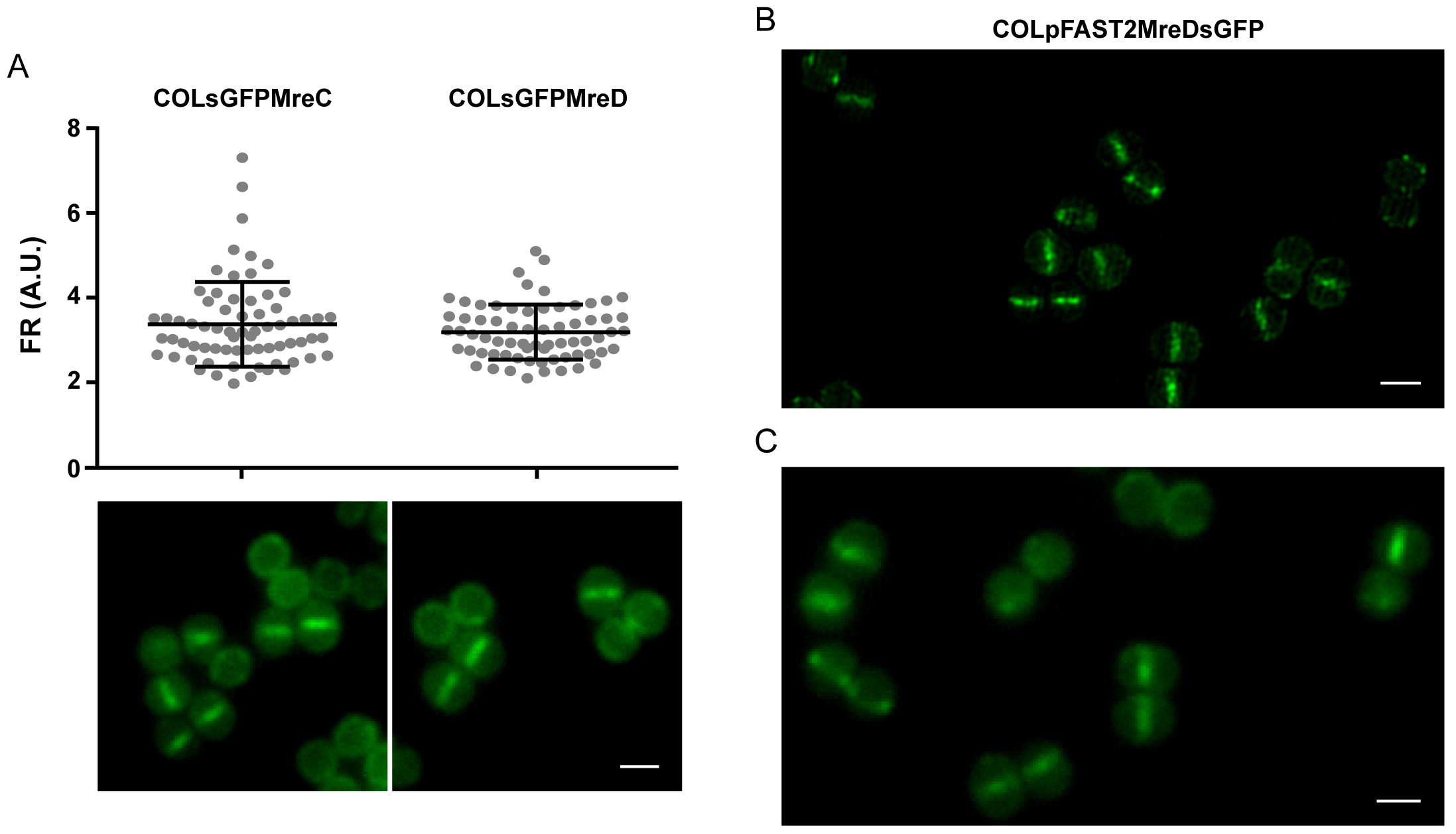

Supplement: S4 Fig — (A) Widefield fluorescence microscopy images of sGFPMreC and sGFPMreD fusions in S. aureus COL. Fluorescence ratio (FR) between the values quantified at the center of the division septum and the fluorescence at the peripheral cell membrane was calculated. A total of 70 cells with closed septa was analyzed for each strain. An FR above two indicates protein enrichment at the septum. (B-C) SR-SIM (B) and Widefield fluorescence microscopy (C) images of COLpFAST2MreDsGFP which expresses MreD-sGFP fusion from the native locus. Scale bars, 1 μm. (TIF) [file pone.0140523.s004.tif]

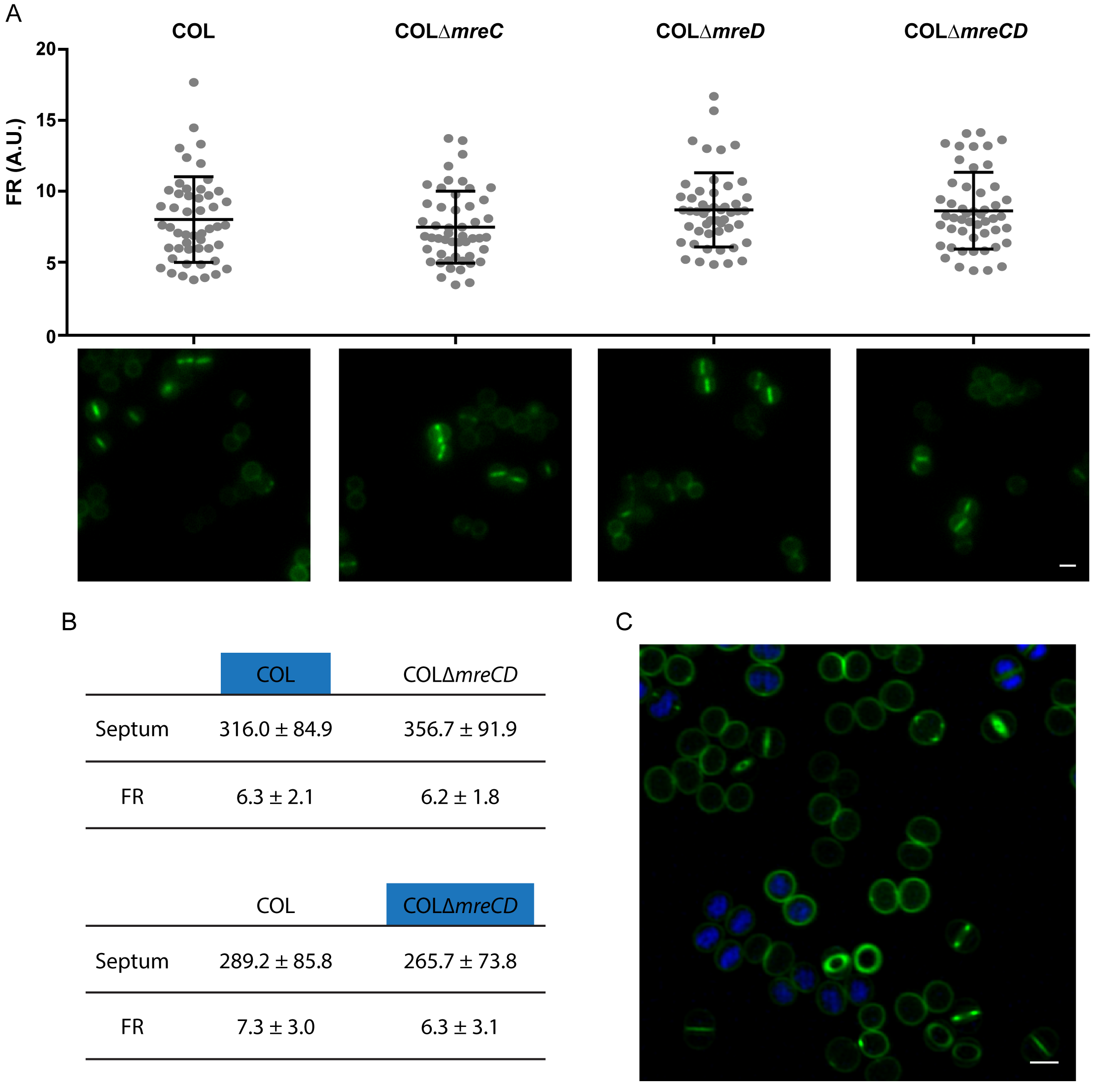

Supplement: S5 Fig — (A) COL, COLΔmreC, COLΔmreD and COLΔmreCD were imaged by widefield fluorescence microscopy after being labeled for 5 minutes with fluorescent derivative of 3-amino-D-alanine, NADA. Incorporation of NADA occurs mainly at the septum but also in the peripheral cell wall. Fluorescence ratio (FR) between the values quantified at the center of the division septum and the fluorescence at the lateral cell wall was calculated. A total of 50 cells with closed septa were analyzed for each strain. The ratios were similar between COL (8.00±3.01) and the mutants ΔmreC (7.47±2.54, p = 0.3445), ΔmreD (8.69±2.62, p = 0.2262) and ΔmreCD (8.63±2.71, p = 0.2754). Scale bar, 1 μm. (B) Septal fluorescence (Septum) and fluorescence ratio (FR) for COL and COLΔmreCD. Cells were labeled with NADA for 5 minutes, mixed together and observed by widefield fluorescence microscopy. To distinguish the two strains, one of them was previously labeled with the DNA dye Hoechst 33342 (shown in blue). The experiment was repeated exchanging the strain with labeled DNA to confirm that Hoechst 33342 labeling does not affect NADA fluorescence. No statistically significant differences were observed between COL parental strain and COLΔmreCD mutant (p>0.05 for all conditions) (C) SR-SIM of COL parental strain (labeled with NADA and Hoechst 33342) and COLΔmreCD (labeled only with NADA). Scale bar, 1 μm. (TIF) [file pone.0140523.s005.tif]

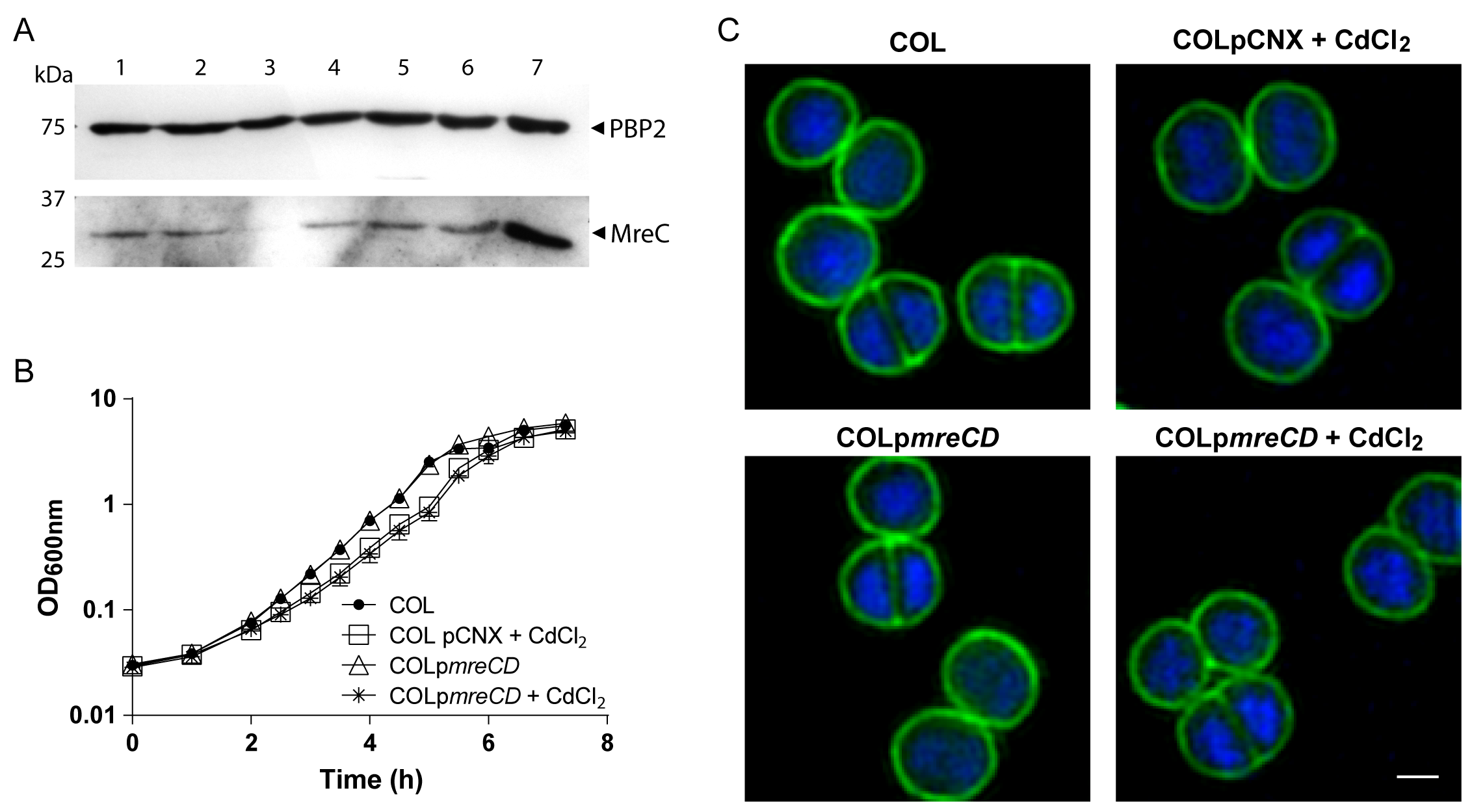

Supplement: S6 Fig — A) Western blot analysis of total protein extracts of COL grown without (1) and with (2) 0.5 mM CdCl2, COLΔmreCD (3), COLpCNX (empty vector) grown without (4) and with (5) 0.5 mM CdCl2 and COLpmreCD grown without (6) and with (7) 0.5 mM CdCl2. MreC detection was performed using an MreC-specific antibody. Overexpression of MreC is observed in COLpmreCD in the presence of the inducer (lane 7). PBP2 was used as internal loading control. (B, C) Growth curves (B) and SR-SIM (C) of COL, COLpCNX grown with 0.5 mM CdCl2 and COLpmreCD grown without and with 0.5 mM CdCl2. Scale bar, 1 μm. Growth curves and morphology of COLpCNX (parental strain with empty vector) and COLpmreCD (overexpressing MreCD), both grown in the presence of cadmium, are indistinguishable. (TIF) [file pone.0140523.s006.tif]
