# Supplementary material for: MreC and MreD Proteins Are Not Required for Growth of Staphylococcus aureus
Source: PLoS One. 2015 Oct 15;10(10):e0140523. doi: 10.1371/journal.pone.0140523 (PMC4607420; doi:10.1371/journal.pone.0140523)
Supplement: S1 File — Information about plasmids, strains and primers used in this study. (DOCX) [file pone.0140523.s007.docx]

Table A - Plasmids used in this study.

| **Plasmids** | **Relevant Genetic Characteristics** | **Source or Ref.** |
| --- | --- | --- |
| pMAD | *E. coli - S. aureus* shuttle vector with a thermosensitive origin of replication for Gram-positive bacteria, Amp^R^, Ery^R^; *lacZ* | [1] |
| pBCB13 | pMAD derivative with up- and downstream regions of spa locus and Pspac-lacI, Amp^R^, Ery^R^; *lacZ* | [2] |
| pMGPII | *S. aureus* replicative plasmid containing *lacI* gene, Amp^R^, Cm^R^ | [3] |
| pMutin4 | *E. coli - S. aureus* shuttle vector; integrative in *S. aureus*, IPTG-inducible P_spac_ promoter, Amp^R^, Ery^R^ | [4] |
| pCNX | Shuttle vector containing a cadmium inducible P_cad_ promoter, Amp^R^ Kan^R^ | [5] |
| pET30a | Cloning vector for producing His tag fusions, Kan^R^ | Novagen |
| pTRC99a-P7 | Vector containing *p7superfastgfp*, Amp^R^ | [6] |
| pSG5082 | *S. aureus* integrative vector for C-terminal GFP fusions, Amp^R^, Ery^R^ | [7] |
| pFAST2 | pSG5082 derivative for C-terminal fusions with *p7superfastgfp* in the native locus; integrative in *S. aureus*, Amp^R^, Ery^R^ | This study |
| pMutin4MreCt | pMUTIN4 with putative ribosome binding site and 463 first nucleotides of mreC under control of the P_spac_ promoter, Amp^R^, Ery^R^ | This study |
| p∆*mreC* | pMAD with *mreC* upstream and downstream regions for constructing a null mutant, Amp^R^, Ery^R^; *lacZ* | This study |
| p∆*mreD* | pMAD with *mreD* upstream and downstream regions for constructing a null mutant, Amp^R^, Ery^R^; *lacZ* | This study |
| p∆*mreCD* | pMAD with *mreCD* upstream and downstream regions for constructing a double null mutant, Amp^R^, Ery^R^; *lacZ* | This study |
| p*mreCD* | pCNX encoding *mreCD* under the control of P_cad_ | This study |
| pETMreCt | pET30a expressing His-MreC | This study |
| psGFPMreC | pBCB13 with s*gfp*-*mreC* fusion under control of the P_spac_ promoter, Amp^R^, Ery^R^; *lacZ* | This study |
| psGFPMreD | pBCB13 with s*gfp*-*mreD* fusion under control of the P_spac_ promoter, Amp^R^, Ery^R^; *lacZ* | This study |
| pFAST2MreDsGFP | pFAST2 with the 483 final nucleotides of *mreD* without the stop codon cloned upstream of and in frame with *sgfp;* Amp^R^, Ery^R^ | This study |

Abbreviations: Kan^R^ – kanamycin resistance; Amp^R^ – ampicillin resistance;

Cm^R^ – Chloramphenicol resistance; Ery^R^ – erythromycin resistance.

Table B - Strains used in this study

| **Strains** | **Relevant Characteristics** | **Source or Ref.** |
| --- | --- | --- |
| ***E. coli*** |  |  |
| DC10B | *dam^+^ dcm^+^ ∆hsdRMS endA1 recA1* | [8] |
| BL21 (DE3) | B F^-^ *dcm ompT hsdS (r_B_^-^m_B_^-^) gal* λ (DE3) | Stratagene |
| ***S. aureus*** |  |  |
| RN4220 | Restriction deficient derivative of NCTC8325-4 | R. Novick |
| NCTC8325-4 | MSSA strain | R. Novick |
| COL | MRSA strain | [9] |
| COL*mreCD*i | COL with *mreCD* operon under the control of P_spac_ promoter and transformed with pMGPII plasmid; Ery^R^, Cm^R^ | This study |
| COL∆*mreC* | COL *mreC* deletion mutant | This study |
| COL∆*mreD* | COL lacking nucleotides 7-380 of *mreD* | This study |
| COL∆*mreCD* | COL lacking *mreC* and first 380 nucleotides of *mreD* | This study |
| NCTC∆*mreC* | NCTC8325-4 *mreC* deletion mutant | This study |
| NCTC∆*mreD* | NCTC8325-4 lacking nucleotides 7-380 of *mreD* | This study |
| NCTC∆*mreCD* | NCTC8325-4 lacking *mreC* and first 380 nucleotides of *mreD* | This study |
| COLpCNX | COL transformed with pCNX empty vector | This study |
| COLp*mreCD* | COL transformed with p*mreCD*oe plasmid with *mreCD* operon under the control of P_cad_ promoter; Kan^R^ | This study |
| COLsGFPMreC | COL *spa*::P_spac_-*sgfp-mreC* | This study |
| COLsGFPMreD | COL *spa*::P_spac_-*sgfp-mreD* | This study |
| COLpFAST2MreDsGFP | COL *mreD*::*mreD*-*sgfp* fusion, Ery^R^ | This study |

Abbreviations: Ery^R^ – erythromycin resistance; Cm^R^ – chloramphenicol resistance.

Table C – Primers used in this study

| **Primer** | **Sequence 5' - 3'** |
| --- | --- |
| MreCt-P1 | CCCAAGCTTGACATAATAGAGGTGTTC |
| MreCt-P2 | CGGGATCCCTTTAGTAACTCTTCCAAC |
| dMreC-P1 | CCCCCCGGGATGAACGTGCATCAGTCCTAAG |
| dMreC-P2 | TATCCCTGCTCACCCAGAACACCTCTATTATG |
| dMreC-P3 | GTTCTGGGTGagcagggataaataatgcg |
| dMreC-P4 | CGGGATCCagtgtatggttgacgatg |
| dMreD-P1 | CCCCCCGGGctggaatgtttgctagtag |
| dMreD-P2 | CAAATTGAATGacgcattatttatccctgc |
| dMreD-P3 | AATAATGCGTcattcaatttgatattattc |
| dMreD-P4 | CGGGATCCgttttttgtagaacttacc |
| dMreCD-P2 | CAAATTGAATGcacccagaacacctctattatg |
| dMreCD-P3 | GTTCTGGGTGcattcaatttgatattattc |
| MreC_*Sma*I_P1 | TCCCCCGGGgacataatagaggtgttctg |
| MreD_*Eco*RI_P2 | CGGAATTCttaccattgacgacgtttc |
| sGFP-*Eco*RV-P1 | cgcggatatcaggaaacagaccatggaattcgagctcggtacacgg |
| sGFP-*Not*I-P2 | GCTTAGCGGCCGCTTAATGGTGATGATGGTGATGGTCGACTTTGTATAG |
| sGFP-*Sma*I-P1 | tcccccgggGGCCAATAAAACTAGGAGGAAATTTAAATGagtaaaggagaagaacttttc |
| sGFPMreC-P2 | ACTTAAGCACGGAGGCGCCGCAGGAtttgtatagttcatccatg |
| sGFPMreC-P3 | ACTATACAAATCCTGCGGCGCCTCCgtgcttaagttttttaaaaataac |
| sGFPMreC-*Xho*I-P4 | CCGCTCGAGttatttatccctgctttc |
| sGFPMreD-P2 | GTGTACGCATGGAGGCGCCGCAGGAtttgtatagttcatccatg |
| sGFPMreD-P3 | ACTATACAAATCCTGCGGCGCCTCCatgcgtacactgtattattttttg |
| sGFPMreD-*Xho*I-P4 | CCGCTCGAGttaccattgacgacgtttc |
| MreDsGFP-*Hind*III-P1 | CCCAAGCTTatagatactgcaattgggc |
| MreD-sGFP-*Bam*HI-P2 | CGGGATCCccattgacgacgtttcatg |
| MreCprot-P1 | CGGGATCCcgttcacaatctcaatcac |
| MreCprot-P2 | CCGCTCGAGttatttatccctgctttcatc |

**References**

1. Arnaud M, Chastanet A, Débarbouillé M. New vector for efficient allelic replacement in naturally gram-positive bacteria. Appl Environ Microbiol. 2004;70:6887–91.

2. Pereira PM, Veiga H, Jorge AM, Pinho MG. Fluorescent reporters for studies of cellular localization of proteins in *Staphylococcus aureus*. Appl Environ Microbiol. 2010;76:4346–53.

3. Pinho MG, Filipe SR, de Lencastre H, Tomasz A. Complementation of the essential peptidoglycan transpeptidase function of Penicillin-Binding Protein 2 (PBP2) by the drug resistance protein PBP2A in Staphylococcus aureus. J Bacteriol. 2001;183:6525–31.

4. Vagner V, Dervyn E, Ehrlich SD. A vector for systematic gene inactivation in *Bacillus subtilis*. Microbiology. 1998;144:3097–104.

5. Monteiro JM, Fernandes PB, Vaz F, Pereira AR, Tavares AC, Ferreira MT, et al. Cell shape dynamics during the staphylococcal cell cycle. Nat Commun. 2015;6:8055.

6. Fisher AC, DeLisa MP. Laboratory evolution of fast-folding green fluorescent protein using secretory pathway quality control. PLoS One. 2008;3:1–7.

7. Pinho MG, Errington J. A divIVA null mutant of *Staphylococcus aureus* undergoes normal cell division. FEMS Microbiol Lett. 2004;240:145–9.

8. Monk IR, Shah IM, Xu M, Tan M, Foster TJ. Transforming the untransformable : application of direct transformation to manipulate genetically *Staphylococcus aureus* and *Staphylococcus epidermidis*. MBio. 2012;3:1–11.

9. Gill SR, Fouts DE, Archer GL, Mongodin EF, Deboy RT, Ravel J, et al. Insights on evolution of virulence and resistance from the complete genome analysis of an early methicillin-resistant *Staphylococcus aureus* strain and a biofilm-producing methicillin-resistant *Staphylococcus epidermidis* strain. J Bacteriol. 2005;187:2426–38.
