# Supplementary material for: MreC and MreD Proteins Are Not Required for Growth of Staphylococcus aureus
Source: PLoS One. 2015 Oct 15;10(10):e0140523. doi: 10.1371/journal.pone.0140523 (PMC4607420; doi:10.1371/journal.pone.0140523)
Supplement: S1 Table — (DOCX) [file pone.0140523.s008.docx]

**S1 Table. Genomic mutations in COL∆*mreD* and COL∆*mreCD* identified by whole genome sequencing by comparison with parental strain COL.**

| **Gene containing indicated mutation** | **Description** | **Nucleotide change** | **Amino acid change** |
| --- | --- | --- | --- |
| **COL∆*mreD*** |  |  |  |
| SACOL_RS09375 | Hypothetical protein | C→A | Gln23Lys |
| **COL∆*mreCD*** |  |  |  |
| SACOL_RS00015 | Plasmid recombination enzyme (pre) | G→A | Ser2Phe |
