# Supplementary material for: MreC and MreD Proteins Are Not Required for Growth of Staphylococcus aureus
Source: PLoS One. 2015 Oct 15;10(10):e0140523. doi: 10.1371/journal.pone.0140523 (PMC4607420; doi:10.1371/journal.pone.0140523)
Supplement: S2 Table — (DOCX) [file pone.0140523.s009.docx]

**S2 Table. Average volume (µm^3^) of *S. aureus* COL, COL∆*mreC*, COL∆*mreD* and COL∆*mreCD* cells at the three phases of the cell cycle.**

| **Strain** | **Phase 1** | **Phase 2** | **Phase 3** |
| --- | --- | --- | --- |
| COL | 0.40 ± 0.07 | 0.60 ± 0.08 | 0.70 ± 0.10 |
| COL∆*mreC* | 0.43 ± 0.09 | 0.60 ± 0.10 | 0.69 ± 0.10 |
| COL∆*mreD* | 0.45 ± 0.08  * | 0.60 ± 0.09 | 0.74 ± 0.13 |
| COL∆*mreCD* | 0.45 ± 0.08  * | 0.63 ± 0.10 | 0.71 ± 0.10 |

* p-value ≤ 0.05 mutants vs. parental strain COL (n=60 cells for each strain)
