# Supplementary material for: MreC and MreD Proteins Are Not Required for Growth of Staphylococcus aureus
Source: PLoS One. 2015 Oct 15;10(10):e0140523. doi: 10.1371/journal.pone.0140523 (PMC4607420; doi:10.1371/journal.pone.0140523)
Supplement: S3 Table — Minimum Inhibitory Concentrations (MICs) of different antibiotics, sodium chloride and hydrogen peroxide and the higher acidic pH value which inhibits cell growth. The values in the table correspond to the results obtained for all the deletion mutants and the parental strain, after 24h incubation, except for oxacillin (48h). (DOCX) [file pone.0140523.s010.docx]

**S3 Table. Susceptibility to antibiotics, osmotic, oxidative and acidic stress agents of COL, COL∆*mreC*, COL∆*mreD* and COL∆*mreCD***.

|  | **MIC** |
| --- | --- |
| **Antibiotics (µg/ml)** |  |
| Oxacillin | 512 |
| Vancomycin | 2 |
| Phosphomycin * | 2000 |
| D-cycloserine | 75 |
| Nalidixic acid | 75 |
| Chloramphenicol | 3.1 |
| Bacitracin | 112.5 |
| Tunicamycin | 150 |
| **Osmotic Stress (M)** |  |
| NaCl (M) | 3 |
| **Oxidative Stress (mM)** |  |
| H_2_O_2_ (mM) | 5 |
| **Acidic Stress** |  |
| pH | 3 - 4 |

* Very poor growth above 125 µg/ml
